# Inflamm-Aging-Related Cytokines of IL-17 and IFN-*γ* Accelerate Osteoclastogenesis and Periodontal Destruction

**DOI:** 10.1155/2021/9919024

**Published:** 2021-08-04

**Authors:** Jingyi Tan, Anna Dai, Lai Pan, Lan Zhang, Zhongxiu Wang, Ting Ke, Weilian Sun, Yanmin Wu, Pei-Hui Ding, Lili Chen

**Affiliations:** ^1^Department of Periodontology, The Second Affiliated Hospital of Zhejiang University, School of Medicine, Hangzhou 310009, China; ^2^Stomatology Hospital, School of Stomatology, Zhejiang University School of Medicine, Clinical Research Center for Oral Diseases of Zhejiang Province, Key Laboratory of Oral Biomedical Research of Zhejiang Province, Cancer Center of Zhejiang University, Hangzhou, Zhejiang 310006, China; ^3^Stomatology Department, Zhejiang Hospital, Hangzhou 310007, China

## Abstract

Periodontal disease (PD), as an age-related disease, prevalent in middle-aged and elderly population, is characterized as inflammatory periodontal tissue loss, including gingival inflammation and alveolar bone resorption. However, the definite mechanism of aging-related inflammation in PD pathology needs further investigation. Our study is aimed at exploring the effect of inflamm-aging-related cytokines of interleukin-17 (IL-17) and interferon-*γ* (IFN-*γ*) on osteoclastogenesis *in vitro* and periodontal destruction in *vivo*. For receptor activator of nuclear factor-*κ*B ligand- (RANKL-) primed bone marrow macrophages (BMMs), IL-17 and IFN-*γ* enhanced osteoclastogenesis, with the expression of osteoclastogenic mRNA (TRAP, c-Fos, MMP-9, Ctsk, and NFATc1) and protein (c-Fos and MMP-9) upregulated. Ligament-induced rat models were established to investigate the role of IL-17 and IFN-*γ* on experimental periodontitis. Both IL-17 and IFN-*γ* could enhance the local inflammation in gingival tissues. Although there might be an antagonistic interaction between IL-17 and IFN-*γ*, IL-17 and IFN-*γ* could facilitate alveolar bone loss and osteoclast differentiation.

## 1. Introduction

Periodontal disease (PD) is a chronic inflammatory disease that occurs in the periodontal support tissue caused by periodontopathic bacteria. It is characterized by gingival inflammation and destruction of alveolar bone [1, 2]. The health of the periodontal tissues is not only directly linked to oral diseases but also implicated in system-wide health, but the pathogenesis of periodontitis is still unidentified. Recent studies have reported that PD is highly prevalent in adults and the disease severity increases with age. More than 64% of adults aged ≥65 suffered from moderate/severe periodontitis, causing a huge economic burden worldwide [3, 4].

Inflamm-aging refers to a proinflammatory state which is chronic, systematic, and controllable in the aging process [5]. The proinflammatory state accelerates the aging process, and in turn, the aging of the body aggravates the development of inflammatory response, thus forming a vicious circle [6]. Inflamm-aging is closely associated with many chronic diseases, such as periodontitis, type 2 diabetes, insulin resistance, atherosclerosis, osteoporosis, and arthritis [7–9]. During aging, chronic periodontal inflammation is often accelerated with the aging of tissues and organs [10].

Recent studies on the mechanisms of inflamm-aging pay attention to oxidative stress, DNA damage, autophagy, and inflammatory cytokines [11–13]. In the cellular immune response, Th1/Th2/Th17/Treg responses and cytokines play important roles in inflamm-aging induced by chronic inflammation [6, 14–17]. During initiation and progression of periodontitis, Th1 cell and mainly cytokine interferon-*γ* (IFN-*γ*) were previously regarded as having key importance in bone loss related to inflammation [18, 19]. However, accumulating pieces of evidences have shown that the inflammation of PD may be dominantly mediated through Th17 cells and its main factor interleukin-17A (IL-17) [20, 21]. We previously found significantly higher levels of IL-17 and IFN-*γ* in plasma, gingival crevicular fluid, and gingival biopsy samples of patients with chronic periodontitis than healthy individuals [22]. However, how IL-17 and IFN-*γ*, as inflamm-aging-related cytokines, regulate the initiation and development of PD remains unclear.

IL-17 has been demonstrated to have multiple effects on the process of bone destruction related to inflammation [23–25]. IL-17 can act on macrophages and synergistically increase the production of inflammatory cytokines, such as tumor necrosis factor-*α* (TNF-*α*), IL-1*β*, and IL-6 [26]. IL-17 can also recruit neutrophils to the inflammatory sites and enhance granulopoiesis to activate neutrophil-mediated inflammation [27]. Our former *in vivo* study showed that there was an increased number of osteoclasts and alveolar bone resorption in IL-17-treated periodontitis rat model [28]. Hence, IL-17 is a potent mediator to bridge aging-related inflammation with alveolar bone.

IFN-*γ* also plays an important part in inflammation and osteoclastogenesis. Since it has a complex role in physiological and pathological conditions, it is difficult to define IFN-*γ* as a proinflammatory or anti-inflammatory cytokine [27]. IFN-*γ* mutant mice challenged with *Porphyromonas gingivalis* showed less alveolar bone loss compared with the sham-operated mice [29], indicating its proresorptive effect on alveolar bone. IFN-*γ* has a dual effect in osteoclasts depending on the level of IFN-*γ*, receptor activator of nuclear factor-*κ*B ligand (RANKL), and the stage of osteoclast differentiation [30]. Several studies reported that IFN-*γ* could exert an inhibitory effect in the early stage of osteoclast differentiation and a stimulatory effect in the late stage of osteoclast maturation [27, 30, 31].

However, the role of IFN-*γ* combined with IL-17 on the process of PD *in vivo* is yet to be elucidated. At present, there are relatively few reports on the mechanism of inflamm-aging in periodontitis, and how to mitigate periodontitis through regulating inflamm-aging is yet pending further study. Hence, the present study is aimed at investigating the effect of inflamm-aging-related cytokines of IL-17 or/and IFN-*γ* on osteoclast differentiation *in vivo*, as well as gingival inflammation and alveolar bone loss in the ligature-induced periodontitis rat model.

## 2. Materials and Methods

### 2.1. Cell Culture and Osteoclast Induction

Bone marrow macrophages (BMMs) were collected as described previously [28]. Briefly, C57BL/6 mice (6-8 weeks old) were anesthetized with sodium pentobarbital (150 mg/kg, intraperitoneally) and then sacrificed through cervical dislocation. The tibiae and femora were dissected with the ends being removed. Then, a syringe was used to flush the marrow cavity with *α*-MEM medium (Gibco, USA) supplemented with 10% FBS (fetal bovine serum, Gibco, USA), 100 U/*μ*l penicillin (TBD Science, China), 100 U/*μ*l streptomycin (TBD Science, China), and 30 ng/ml macrophage colony-stimulating factor (M-CSF; R&D, Minneapolis, MN, USA). The mixed cells were incubated at the environment of 37°C and 5% CO_2_ for 3 days. The adherent cells were considered as BMMs and used for the following *in vitro* studies.

To induce osteoclasts, BMMs were seeded in 48-well plates at the density of 3 × 10^4^ cells/well in the presence of M-CSF (30 ng/ml) and RANKL (20 ng/ml, R&D, Minneapolis, MN, USA). Different concentrations of IL-17 (0.1, 1, and 10 ng/ml, PeproTech, Rocky Hill, NJ, USA) and IFN-*γ* (0.02, 0.2, and 2 ng/ml, PeproTech, Rocky Hill, NJ, USA) were added on the third day or the sixth day after seeding. The culture media were changed every other day.

### 2.2. Ligature-Induced Periodontitis Rat Model

A total of 75 eight-week-old Sprague-Dawley rats (200 ± 20 g) were obtained from the China Experimental Animal Center (Hangzhou, China). The rats were housed in individual cages under the temperature of 21 ± 2°C with 12 h light-dark cycles and humidity of 50 ± 5%. They were fed with high-glucose laboratory food and high-glucose tap water *ad libitum*. The protocol of this rat experiment was approved by the Animal Ethics Committee of the Second Affiliated Hospital of Zhejiang University School of Medicine (No. 2017-052).

Subjects were divided randomly into 5 groups: NC (normal control) group, NS (normal saline) group, IL-17 group, IFN-*γ* group, and IL-17+IFN-*γ* group. Each group included five rats. To establish an experimental periodontitis rat model, as previously described [28], after the anesthetization procedure with sodium pentobarbital (40 mg/kg) via intraperitoneal injection, 2 mm orthodontic ligatures (West Lake Barr, Hangzhou West Lake Biomaterials Co., China) were fixed around the cervical margins around the bilateral maxillary first molars. In the NC group, sham treatment was applied. Afterward, the rats received injection of 20 *μ*l normal saline, 20 *μ*l IL-17 (5 *μ*g/ml), or/and IFN-*γ* (1 *μ*g/ml) into the soft tissue around the maxillary bilateral first molars under anesthesia every other day, respectively. The rats in the NC group did not receive any injection. After 1, 2, and 4 weeks, rats were humanely sacrificed under anesthesia.

### 2.3. Histological Observation

The right sides of the maxillary first molars and the surrounding tissues of the 1-week and 2-week samples were harvested and fixed in 4% phosphate-buffered paraformaldehyde (Boster Biological Technology, Wuhan, China) (pH = 7.2) for 48 h and then decalcified in 10% EDTA solution for 8 weeks. The decalcified samples were dehydrated in ethanol and embedded in paraffin. Subsequently, the mesiodistal slices with thickness of 5 *μ*m were obtained and stained with hematoxylin and eosin (HE). The number of inflammatory cells, mainly neutrophils, and lymphocytes in the interproximal regions between the first and second molars was counted according to their morphologies. The mean number of five randomly selected fields at 400x magnification was used for each section.

### 2.4. Immunohistochemistry and Tartrate-Resistant Acid Phosphatase (TRAP) Staining

Dewaxed and rehydrated sections were treated with 3% hydrogen peroxide for 15 min to block endogenous peroxidase activity. After washing in PBS, sections were incubated with primary polyclonal antibodies against IL-6 (1 : 100; Proteintech, Chicago, IL, USA), IL-1*β* (1 : 100; Proteintech, Chicago, IL, USA), or TNF-*α* (1 : 100; Proteintech, Chicago, IL, USA) overnight at 4°C. The sections were then washed and treated with secondary antibodies (Boster Biological Technology, Wuhan, China). Afterward, these sections were stained using a 3,3′-diaminobenzidine kit (Boster Biological Technology, Wuhan, China) and then counterstained with hematoxylin.

Osteoclasts were classified by the specific marker tartrate-resistant acid phosphatase (TRAP) using a kit from Sigma (St. Louis, MO, USA). Active osteoclasts were identified when TRAP-positive cells contained three or more nuclei.

IL-6-positive, IL-1*β*-positive, and TNF-*α*-positive cells and active osteoclasts in the interradicular regions of the first molars were counted at 400x magnification. The mean number of the five randomly selected fields was recorded for each section, and three sections of each rat were counted.

### 2.5. Micro-Computed Tomography (Micro-CT) Scanning

The 4-week samples of molars were scanned using a micro-CT scanning machine (Scanco Medical AG, Bassersdorf, Switzerland). The micro-CT parameters were set as follows: pixel size, 10 × 10 *μ*m; slice thickness, 10 *μ*m; voltage, 70 kV; and electrical current, 200 *μ*A.

The images were reconstructed to generate three-dimensional (3D) models. Bone loss was measured as previously described [32], which was the distance from the cemento-enamel junction (CEJ) to the alveolar bone crest (ABC) along the buccal and lingual long axis of the maxillary first molars. Six sites were detected per tooth for each rat.

A circle with fixed diameter was selected as the observation area between the mesial and distal root of the first molar on the transverse section. The interradicular regions of the maxillary first molars were selected for bone volume per tissue volume (BV/TV) analysis.

### 2.6. Reverse Transcription-Quantitative Polymerase Chain Reaction (RT-qPCR) Analysis

RT-qPCR analysis was applied to evaluate the gene expression of osteogenic markers in BMMs and gingival samples. Cells were harvested using TRIzol reagent (Invitrogen; Thermo, Waltham, MA, USA) two days after adding IL-17 or/and IFN-*γ*. Gingival tissue with a width of 2 mm below the gingival margin of the left first molars of 2-week and 4-week samples was excised and grinded. Total cellular RNA was extracted following treatment with TRIzol reagent on ice. A total of 1 *μ*g RNA was reverse-transcribed to generate single-stranded cDNA using the PrimeScript Reverse Transcription Master Mix Kit (Takara, Otsu, Japan). The reverse transcription protocol was as follows: 37°C for 15 min, 85°C for 5 sec and terminating at 4°C. The expression level of target genes was quantified using a SYBR Premix Ex Taq™ II kit (Takara, Otsu, Japan) on the StepOnePlus Real-Time PCR System (Applied Biosystems; Thermo, Waltham, MA, USA). The primer sequences are listed in Table S1.

The amplification procedure was performed for 40 cycles at 95°C (30 seconds), 95°C (5 seconds), and 60°C (30 seconds). The relative expression level of target genes was calculated by the relative quantitative method (2^-*ΔΔ*Cq^) and normalized to the mouse GAPDH gene.

### 2.7. Western Blotting Analysis

Western blotting analysis was applied to evaluate the protein expression in BMMs which were harvested by cell lysate buffer (Thermo Fisher Scientific, USA) two days after adding IL-17 or/and IFN-*γ*. After being quantified by bicinchoninic acid kit (Thermo Fisher Scientific, USA), equal amounts of total protein (20 *μ*g) were resolved by SDS-PAGE on a 10% gel and transblotted onto the polyvinylidene fluoride membrane (EMD Millipore, Billerica, MA, USA). The membranes were then blocked with 5% skim milk (BD Difco, USA) for 1 h at room temperature. Anti-GAPDH (1 : 1000, Fdbio Science, China), c-Fos (1 : 1000, Proteintech, Chicago, IL, USA), and MMP-9 (1 : 1000, Abcam) were incubated overnight at 4°C. Membranes were washed and incubated with alpaca anti-rabbit antibody (1 : 50000, HuaBio, China) and peroxidase-conjugated goat anti-mouse antibody (1 : 5000, Fudebio, China) at room temperature for 1 h. After washing, immunoreactive bands were visualized using an ECL kit (Fudebio, China) using chemiluminescence imaging apparatus (Tanon 5200 Multi, Tanon, China).

### 2.8. Statistical Analysis

Each experiment was performed in triplicate. All quantitative data are presented as mean ± standard deviation. Statistical analyses between groups were performed by one-way analysis of variance (ANOVA), followed by Tukey-*t*-test using GraphPad Prism 5 (GraphPad Software, La Jolla, CA, USA). Statistically significant difference was considered when *p* < 0.05.

## 3. Results

### 3.1. IL-17 and IFN-*γ* Enhance Osteoclast Differentiation of Mouse Bone Marrow Macrophages

**Figure 1:**
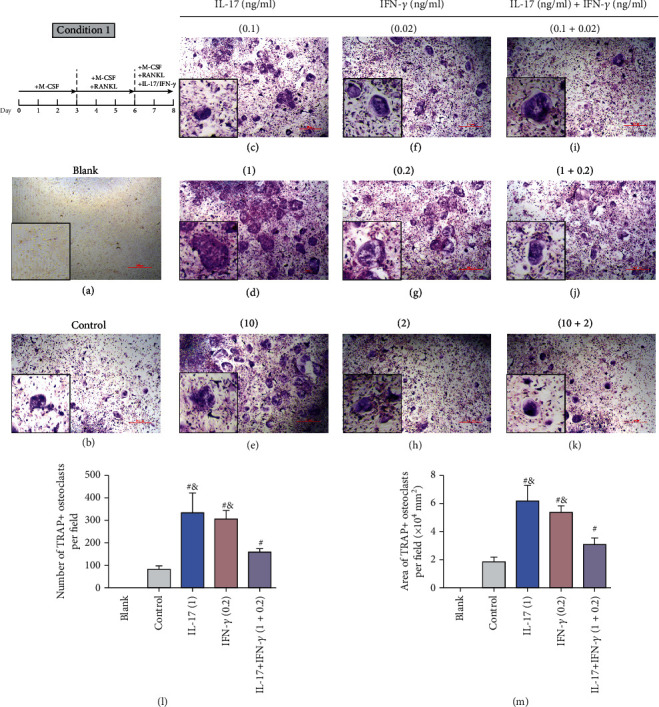
Effect of IL-17 or/and IFN-*γ* on differentiation of preosteoclasts. (a–k) In condition 1, BMMs were cultured with M-CSF (30 ng/ml) for 3 days and then with 20 ng/ml RANKL for another 3 days. On day 6, IL-17 or/and IFN-*γ* of different concentrations was added. (l, m) The results of TRAP staining of each group are presented (scale bar = 500 *μ*m). Osteoclasts were recognized as TRAP-positive multinucleated cells (≥3 nuclei). (l) The number of TRAP-positive osteoclasts and (m) the area of TRAP-positive osteoclasts were counted and presented (mean ± standard deviation, *n* = 3). ^#^*p* < 0.05 compared with the control group; ^&^*p* < 0.05 compared with the IL-17+IFN-*γ* group.

To evaluate the effects of IL-17 or/and IFN-*γ* on osteoclast differentiation, BMMs were cultured with different concentrations of IL-17 (0.1, 1, and 10 ng/ml) or/and IFN-*γ* (0.02, 0.2, and 2 ng/ml). At the setting of condition 1, IL-17 or/and IFN-*γ* were added on day 6 when some of the BMMs have been preliminarily induced into minor multinucleated cells. The formation of TRAP-positive multinucleated cells is the hallmark of osteoclast formation. The number of osteoclasts was counted, and the area of osteoclasts was calculated (Figures 1(l) and 1(m) and Figure S1). The result showed significantly larger TRAP-positive cells when adding IL-17 or IFN-*γ* compared to the control group. The groups of median concentration of IL-17 (1 ng/ml) and IFN-*γ* (0.2 ng/ml) showed the best differentiation capacity of BMMs. However, when adding both IL-17 and IFN-*γ*, the formation of giant mature osteoclasts was significantly inhibited (Figures 1(i)–1(k)) compared with the respective groups of IL-17 or IFN-*γ* alone.

Interestingly, when IL-17 or IFN-*γ* was added together with RANKL at the beginning (condition 2), the number and area of TRAP-positive cells were not significantly upregulated compared to the control group (Figure S2), indicating that the early exposure to IL-17 or/and IFN-*γ* might attenuate the effect of RANKL on the differentiation of osteoclasts.

### 3.2. IL-17 and IFN-*γ* Upregulated the Expression of Osteoclastogenic mRNA and Protein *In Vitro*

**Figure 2:**
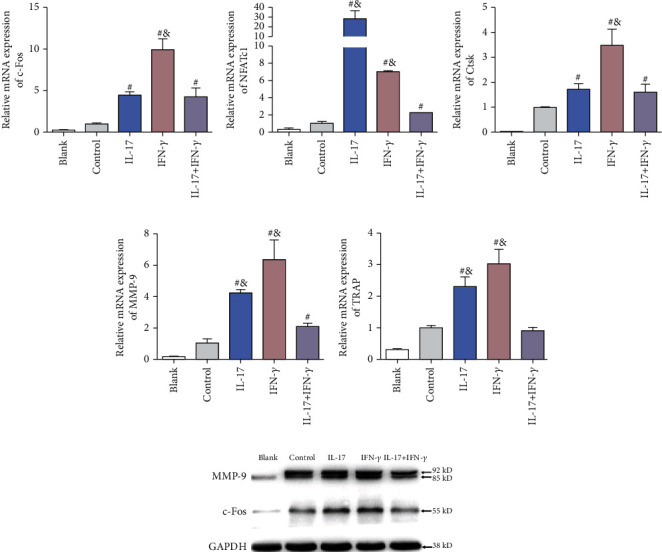
Effects of IL-17 (1 ng/ml) or/and IFN-*γ* (0.2 ng/ml) on the expression of osteoclastogenic genes *in vitro*. The mRNA expression of osteoclast-related genes (a) c-Fos, (b) NFATc1, (c) Ctsk, (d) MMP-9, and (e) TRAP was detected with different concentrations of IL-17 or/and IFN-*γ* using RT-qPCR. Data were standardized to GAPDH expression and shown as a fold change relative to the control group (mean ± standard deviation, *n* = 3). ^#^*p* < 0.05 compared with the control group; ^&^*p* < 0.05 compared with the IL-17+IFN-*γ* group.

In an attempt to explore the explanation for the stimulatory effect of IL-17 and IFN-*γ* on osteoclastogenesis of BMMs, the osteoclastogenic gene expression was analyzed using RT-qPCR, including c-Fos, nuclear factor of activated T cells 1 (NFATc1), cathepsin K (Ctsk), matrix metallopeptidase 9 (MMP-9), and TRAP. The mRNA expression of c-Fos, NFATc1, Ctsk, MMP-9, and TRAP was significantly upregulated in the presence of IL-17 or IFN-*γ* compared to the control group (Figure 2, Figure S3). Consistent with the result of TRAP staining, the group of IL-17 (1 ng/ml) and IFN-*γ* (0.2 ng/ml) showed the strongest elevation of these osteoclastogenic genes. However, the combined addition of IL-17 and IFN-*γ* displayed significantly lower mRNA level of osteoclastogenic genes, suggesting the antagonistic effect between IL-17 and IFN-*γ*.

As for the results of Western blotting analysis, the protein level of c-Fos and MMP-9 was significantly increased in the presence of IL-17 (1 ng/ml) or IFN-*γ* (0.2 ng/ml) compared to the control group (Figure 2). Similarly, the combined use of IL-17 and IFN-*γ* showed decreased proosteoclastogenic effect.

### 3.3. IL-17 and IFN-*γ* Induced Local Gingival Inflammation in Experimental Periodontitis Rat Model

**Figure 3:**
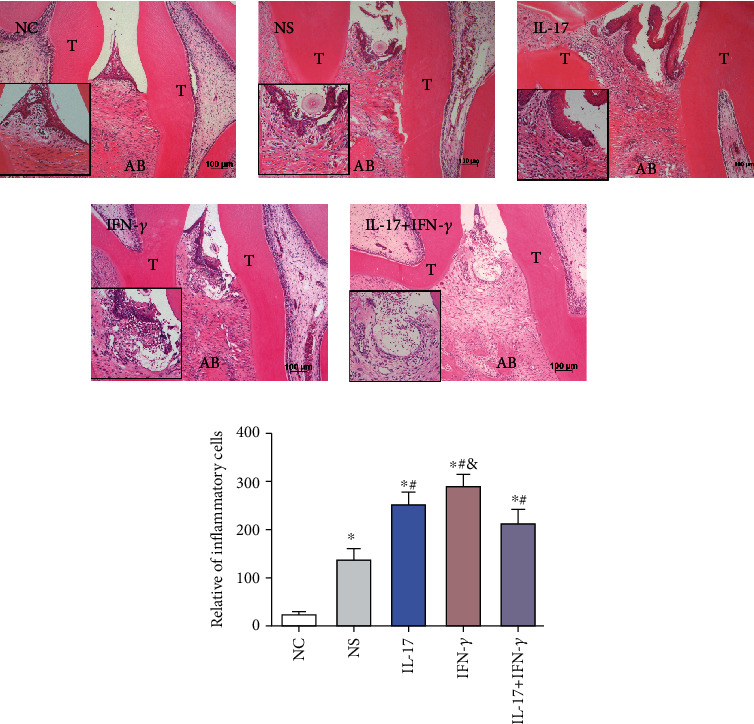
Role of IL-17 (5 *μ*g/ml) or/and IFN-*γ* (1 *μ*g/ml) on local gingival inflammation in an experimental periodontitis rat model. (a) HE staining showed the periodontal morphology in the interradicular regions of the first maxillary molars of the NC, NS, IL-17, IFN-*γ*, and IL-17+IFN-*γ* groups (scale bar = 100 *μ*m). (b) Quantitative analysis was performed on the number of inflammatory cells of each group (mean ± standard deviation, *n* = 5). ^∗^*p* < 0.05 compared with the NC group, ^#^*p* < 0.05 compared with the NS group, and ^&^*p* < 0.05 compared with the IL-17+IFN-*γ* group. T: tooth; AB: alveolar bone.

To investigate the effect of IL-17 and/or IFN-*γ* on the periodontal tissue *in vivo*, we established a rat model of ligature-induced periodontitis and the respective cytokines were locally injected every two days. After 2 weeks, HE staining showed normal gingiva structure with well-aligned periodontal ligament fiber orientation and few inflammatory cells in the NC group (Figure 3). However, in the ligature-induced groups (NS group, IL-17 group, IFN-*γ* group, and IL-17+IFN-*γ* group), the periodontal ligament fibers were disorderly arranged and the alveolar bone lost by varying degrees. Histology results confirmed the typical periodontitis symptoms in the experimental periodontitis group. Many inflammatory cells mainly polymorphonuclear leukocytes invaded the connective tissue as well as the epithelium layer in the NS group, the IL-17 group, the IFN-*γ* group, and the IL-17+IFN-*γ* group compared to the NC group (Figure 3). After calculating the inflammatory cells, the IL-17group and the IFN-*γ* group presented significantly more inflammatory cells than the NS group (*p* < 0.05). Nevertheless, the combined injection of IL-17 and IFN-*γ* resulted in less inflammation than groups that were injected IL-17 or IFN-*γ* solely (*p* < 0.05).

### 3.4. IL-17 and IFN-*γ* Enhance the Proinflammatory Cytokine Expression in Interradicular Regions of the First Maxillary Molars and in Gingival Tissues *In Vivo*

**Figure 4:**
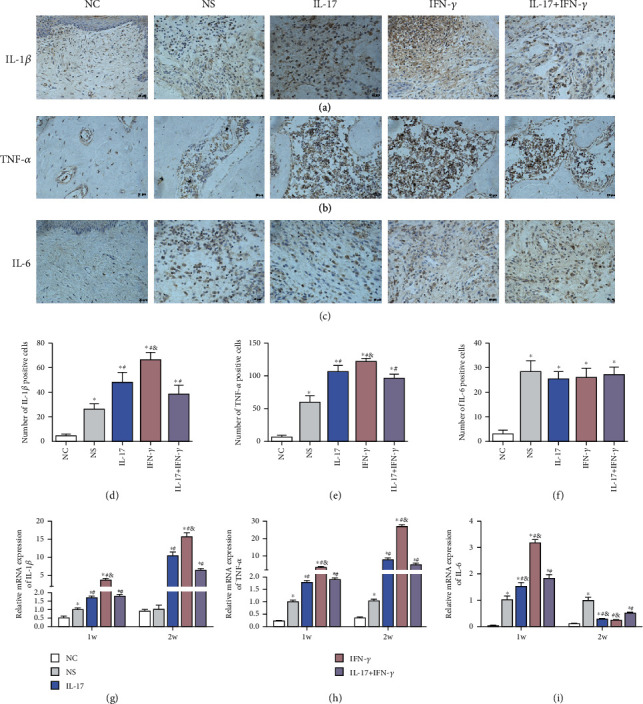
Effect of IL-17 (5 *μ*g/ml) or/and IFN-*γ* (1 *μ*g/ml) on proinflammatory cytokine expression *in vivo*. Immunohistochemical staining was used to examine the level of (a) IL-1*β*, (b) TNF-*α*, and (c) IL-6 expression in gingival tissues (scale bar = 20 *μ*m). Quantitative analysis results of (d) IL-1*β*-, (e) TNF-*α*-, and (f) IL-6-positive cells are shown. The mRNA level of (g) IL-1*β*, (h) TNF-*α*, and (i) IL-6 in gingival tissues after 1 week and 2 weeks was detected by RT-qPCR. Data were presented by mean ± standard deviation, *n* = 5. ^∗^*p* < 0.05 compared with the NC group, ^#^*p* < 0.05 compared with the NS group, and ^&^*p* < 0.05 compared with the IL-17+IFN-*γ* group.

As the immunohistochemistry results show (Figures 4(a)–4(c)), the immunoreactive cells of IL-1*β*, TNF-*α*, and IL-6 were characterized by brown-colored cytoplasm in immunohistochemical staining. The cytokines IL-6 and IL-1*β* were found having higher expression in fibroblasts and inflammatory cells in the ligature-induced gingival zone, while TNF-*α* was found having higher expression in the inflammatory cells in the bone resorption zone. Compared with the NC group, IL-1*β*, TNF-*α*, and IL-6 were observed significantly increased in the NS, IL-17, IFN-*γ*, and IL-17+IFN-*γ* groups (*p* < 0.05). IL-1*β* and TNF-*α* were found having significantly higher expression in the IL-17 and IFN-*γ* groups than in the NS group (*p* < 0.05), but with significantly lower expression in the IL-17+IFN-*γ* group than in the IL-17 or IFN-*γ* group (*p* < 0.05). However, the IL-6 results were comparable among the ligature-induced groups (*p* > 0.05).

The mRNA expression of proinflammatory cytokines (IL-1*β*, TNF-*α*, and IL-6) was examined by RT-qPCR (Figures 4(g)–4(i)). After the periodontitis model establishment for 1 week, significantly increased mRNA expression of IL-1*β*, TNF-*α*, and IL-6 was observed in the IL-17 and IFN-*γ* groups than in the NS group (*p* < 0.05). However, after combined injection of IL-17 and IFN-*γ*, these proinflammatory cytokines showed lower expression than the IL-17 group or the IFN-*γ* group (*p* < 0.05). After 2 weeks, the same tendency of IL-1*β* and TNF-*α* expression among groups was observed in gingival samples. On the contrary, IL-6 level was decreased, and the IL-17 and IFN-*γ* groups showed even lower IL-6 expression than the NS group (*p* < 0.05).

### 3.5. IL-17 and IFN-*γ* Facilitate Alveolar Bone Loss and Osteoclast Differentiation in Experimental Periodontitis Rat Model

**Figure 5:**
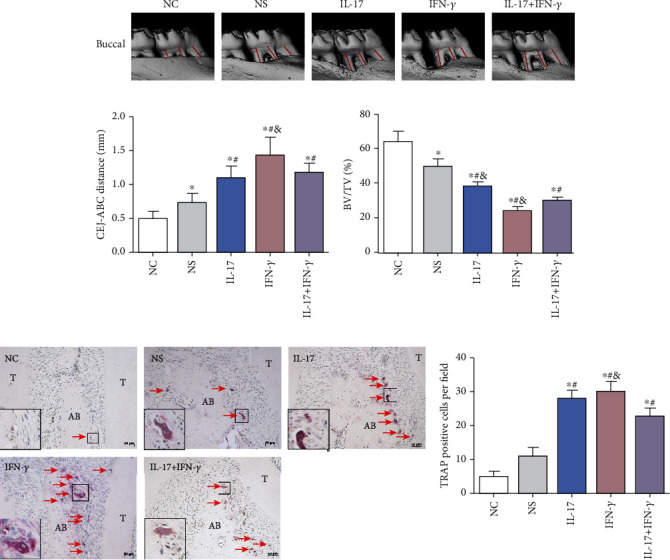
Effect of IL-17 (5 *μ*g/ml) or/and IFN-*γ* (1 *μ*g/ml) on alveolar bone loss and osteoclast differentiation. (a) Reconstructed micro-CT images showed the buccal view of the first maxillary molars. The distance from CEJ to ABC indicated bone loss, shown by the red lines. Quantitative analysis results of the (b) CEJ-ABC distance and (c) BV/TV are shown as mean ± standard deviation. (d) TRAP staining was used to evaluate the formation of osteoclasts (red arrows) for the NC, NS, IL-17, IFN-*γ*, and IL-17+IFN-*γ* groups (scale bar = 50 *μ*m). (e) Quantitative analysis was performed on the number of TRAP-positive multinucleated cells in the interradicular regions of the first maxillary molars (mean ± standard deviation, *n* = 5). ^∗^*p* < 0.05 compared with the NC group, ^#^*p* < 0.05 compared with the NS group, and ^&^*p* < 0.05 compared with the IL-17+IFN-*γ* group. ABC: alveolar bone crest; CEJ: cemento-enamel junction; BV: bone volume; TV: tissue volume; T: tooth; AB: alveolar bone.

We used micro-CT to further detect the alveolar bone level around teeth after 4-week induction, and the reconstructed three-dimensional images of these groups are presented in Figure 5(a). The CEJ-ABC results of the first maxillary molar were 0.49 ± 0.1 mm, 0.74 ± 0.12 mm, 1.09 ± 0.16 mm, 1.42 ± 0.27 mm, and 1.17 ± 0.13 mm for the NC, NS, IL-17, IFN-*γ*, and IL-17+IFN-*γ* groups, respectively. The cytokine injection groups showed more alveolar bone loss around the first maxillary molar than the NS group. In addition, the combined injection of IL-17 and IFN-*γ* resulted in significantly less bone loss than the IFN-*γ* group (*p* < 0.05) but was comparable with the IL-17 group (*p* > 0.05).

When comparing the bone structure parameters, the ligature-induced groups exhibited decreased bone volume per tissue volume (BV/TV), and the injection of IL-17 and IFN-*γ* could accelerate this tendency.

The formation of TRAP-positive multinucleated cells is regarded as the hallmark of osteoclast differentiation. TRAP staining exposed more TRAP-positive cells in the ligature-induced groups than the NC group, as reflected by the increased quantity of multinucleated giant cells. After calculating their number in the interdental zone between the first molar and the second molar, the results showed more TRAP-positive multinucleated cell formation in the IL-17 group (27.67 ± 2.08), the IFN-*γ* group (30.00 ± 2.65), and the IL-17+IFN-*γ* group (22.33 ± 2.52) than in the NC group (4.67 ± 1.53) (*p* < 0.05) (Figures 5(d) and 5(e)).

## 4. Discussion

Inflammation is a complex response to pathogen infection and tissue injury in host immunity. Various mechanisms such as autophagy and reactive oxygen species (ROS) mediate the production of proinflammatory factors, and the excessive proinflammatory response leads to inflammatory aging [11, 12]. Periodontal pathogen-induced host immune response and the overexpression of cytokines exert a profound impact on aggravating gingival inflammation and activating bone destruction [33]. Despite increasing awareness of periodontitis impact on general health, limited progress has been made in understanding the immunological mechanisms to control aging-related inflammation. Our data demonstrated that the use of IL-17 or IFN-*γ* could enhance osteoclastogenesis *in vitro* and exaggerate gingival inflammation and alveolar bone loss *in vivo*. However, there might be an antagonistic interaction between IL-17 and IFN-*γ*.

In the inflamm-aging process, given the vital role of the innate immune system in maintaining periodontal tissue homeostasis through regulated production of proinflammatory cytokines, excessive inflammation can impact bone metabolism. Our *in vitro* study showed that IL-17 and IFN-*γ* could increase the number and the area of TRAP-positive multinucleated cells as well as the expression of osteoclastogenic markers (c-Fos, NFATc1, Ctsk, MMP-9, and TRAP), indicating their proosteoclastogenic capability. However, early exposure to RANKL could render BMMs resistant to IL-17 and IFN-*γ* and result in less formation of mature osteoclasts, indicating the timing of the occurrence of RANKL played an essential role. Both IL-17 and IFN-*γ* played a stimulatory role on the RANKL-induced preosteoclasts rather than uninduced BMMs. Our previous research showed that IL-17 could enhance osteoclast differentiation via activation of autophagy [28]. In this study, we found that simultaneous treatment of RANKL and IL-17 could reverse the proosteoclastogenic capability of IL-17. It is reported that IL-17A could suppress the expression of osteoclastogenic proteinases and osteoclast differentiation, which is attributed to the high concentrations of IL-17A [34]. Huang et al.'s finding also showed that IFN-*γ* had a dual effect on osteoclastogenesis in mouse macrophage cell line RAW264.7, depending on the stage of osteoclast precursors when IFN-*γ* was added [31]. In the early stage when macrophages have not encountered sufficient RANKL, IFN-*γ* potently induces macrophage activation; with pretreatment of RANKL that induces macrophages into preosteoclasts, IFN-*γ* could enhance the maturation of preosteoclasts [31]. Of note, the effect of IL-17 and IFN-*γ* on osteoclastogenesis depends on their concentration due to complex signal pathway regulation. In our study, IL-17 (1 ng/ml) and IFN-*γ* (0.2 ng/ml) showed the most significant effect for osteoclastogenesis, but the trend is not dose dependent. Some researchers reported similar results. Adamopoulos et al. [35] found that exposure to IL-17A could promote peripheral blood mononuclear cells (hPBMCs) to differentiate into functional osteoclasts, while this effect is not dose dependent. Among the concentration setting of 0.1, 1, 10, and 100 ng/ml, 1 ng/ml of IL-17A showed the most significant induction [35]. Another study by Ke et al. [36] found that a low concentration of IL-17A (0.5 ng/ml) could facilitate autophagy of osteoclast precursors via the RANKL-JNK signaling pathway, thus enhancing RANKL-induced osteoclastogenesis. However, treatment with a high concentration of IL-17A (5-50 ng/ml) might inhibit autophagy and decrease osteoclast formation [36]. As for the role of IFN-*γ* on osteoclastogenesis, Huang et al. [31] showed that after pretreatment of RANKL (50 ng/ml), low concentration of IFN-*γ* (0.1, 1.0 ng/ml) could enhance osteoclast formation. However, higher concentration of IFN-*γ* (10, 100 ng/ml) markedly inhibited osteoclastogenesis [31]. Moreover, Kim et al. [37] found that IFN-*γ* (0, 0.1, 0.5, and 1.0 ng/ml) could enhance osteoclast fusion at a dose-dependent manner when BMMs were treated with M-CSF (20 ng/ml) and RANKL (50 ng/ml) for 3 days before IFN-*γ* addition. If IFN-*γ* (1 ng/ml) was added together with M-CSF and RANKL, it could exert an inhibitory effect, indicating that IFN-*γ* could induce the differentiation of RANKL-induced BMMs into multinucleated osteoclasts [37].

The effect of IL-17 and IFN-*γ* on periodontal gingival tissues and alveolar bone suggests their proresorption roles for periodontitis *in vivo*, which might partly be explained by their stimulatory effect on IL-1*β*, TNF-*α*, and IL-6. Salvioli et al. found that the expression levels of TNF-*α*, IL-1, and IL-6 and other cytokines increased with aging [38]. These cytokines, also known as senescence-associated secretory phenotype (SASP), are important pathogenic factors of inflamm-aging. Emerging experimental and clinical studies have demonstrated that TNF-*α*, IL-1*β*, and IL-6 are associated with the initiation and progression of periodontitis [39–42]. In this study, two weeks after the model establishment, the soft tissue lesions in the induced groups were characterized by hyperplasia and migration of gingival epithelium, inflammatory cell infiltration into gingival epithelium and periodontal ligament, and disruption of the periodontal ligament. Besides, local injection of IL-17 and IFN-*γ* could enhance the expression of mRNA and protein of TNF-*α*, IL-1*β*, and IL-6. Notably, the mRNA level of TNF-*α* and IL-1*β* of 2 weeks was higher than that of 1 week, while IL-6 mRNA expression showed decreased tendency. The possible reason might be that IL-6 played a proinflammatory and preosteoclastogenic role prior to TNF-*α* and IL-1*β*. This is in line with Pesic et al.'s findings that the level of TNF-*α* did not show significant changes during the early phase of fracture healing, while IL-6 increased statistically on the first day after intervention [43]. We speculated that the stimulating effects of IL-17 and IFN-*γ* on the expression of TNF-*α* and IL-1*β* might last longer than IL-6.

In parallel, both the height and density of alveolar bone were significantly decreased in the rat model after the administration of IL-17 or IFN-*γ*. Furthermore, the TRAP staining result of alveolar bone showed an increased number of multinucleated osteoclasts in the IL-17 and IFN-*γ* groups. Some *in vivo* studies found that the knockout of IL-17 or its receptor does not affect osteoclast numbers or bone mass in uninduced animal models but exerts a proosteoclastogenic role on parathyroid hormone- (PTH-) induced, ovariectomy- (OVX-) induced bone loss [44, 45]. These results indicated that IL-17 might only affect bone under inflammatory conditions rather than normal physiological conditions. As for IFN-*γ*, the knockout of IFN-*γ* or its receptors in animal models could induce bone loss [46], while the effect of IFN-*γ* administration on bone mass depends on the dosage, frequency, and course [47]. These contradictory results might be attributed to the predominant effect on osteoclastogenesis, the balance between osteoblasts and osteoclasts, and the interaction between bone and immune system. Further researches are required to reveal the complicated roles of IL-17 and IFN-*γ* in different pathophysiological conditions.

Both in vivo and in vitro results indicated the antagonistic interaction between IL-17 and IFN-*γ*. As reported by Tu et al., both IL-17 and IFN-*γ* are required in combination for autoreactive T cells to cause severe damage in autoimmune gastritis [48]. IL-17 and IFN-*γ* also have the potential to induce proteoglycan-induced arthritis (PGIA), but IFN-*γ* suppresses IL-17 production, revealing that the effect of Th17 cells in PGIA requires alleviating IFN-*γ*-mediated IL-17 inhibition [49]. Therefore, we speculate that IL-17 production was weakened after IFN-*γ* interferes with the rat periodontitis model, and the proinflammatory role played by IL-17 decreased. Meanwhile, it should be noted that IL-17 in PGIA is pathogenic only when IFN-*γ* is ablated or reduced, indicating that IL-17 can play a proinflammatory role in PGIA if it is released from the inhibitory effect of IFN-*γ* [49]. This also suggested that IFN-*γ* might still inhibit the proinflammatory effect of IL-17 even though IL-17 and IFN-*γ* exist as proinflammatory factors during the progression of periodontitis.

We previously found that the expression of TRAF6, p-ERK, and p-p38 was all significantly upregulated in osteoclast precursor cells after IL-17 intervention, which suggested that the TRAF6/ERK/p38 signaling pathway might be involved in IL-17-mediated osteoclast differentiation [50]. In Li et al.'s study [51, 52] about the interaction between IL-17 and IFN-*γ* on the growth of mouse hepatoma Hepa1-6 cells (HCC), IL-17 or IFN-*γ* alone could significantly activate p38 MAPK and ERK1/2. It is noteworthy that the phosphorylation levels of p38 MAPK and ERK1/2 were significantly reduced after the combined intervention of IL-17 and IFN-*γ*, indicating that IFN-*γ* might antagonize the effect of IL-17 through the p38 MAPK/ERK1/2 signaling pathway. Considering our finding that there might be an antagonistic effect between IL-17 and IFN-*γ*, we speculated that this antagonistic effect might attribute to the regulation of p38MAPK and ERK signaling pathways during osteoclast differentiation, while the specific mechanism needs further verification.

## 5. Conclusion

In summary, the mechanism of inflamm-aging in the development of periodontitis is still in its nascent stages. Based on the key role of inflammatory cytokines in the process of inflamm-aging, our study revealed that IL-17 and IFN-*γ* could enhance osteoclastogenesis *in vitro*, while their proosteoclastogenic effect only played on the RANKL-primed BMMs. In ligament-induced experimental periodontitis rat model, IL-17 and IFN-*γ* played a proinflammatory role in gingival tissues and proresorption role in alveolar bone. However, there might be an antagonistic interaction between IL-17 and IFN-*γ*. These findings contribute to a better understanding of inflamm-aging cytokines related to alveolar bone resorption in the initiation and progression of PD, thus providing insight into the potential clinical therapeutic targets for PD.

## Data Availability

Data will be available from authors on request.

## Abbreviations

PD: Periodontal disease
IL-17: Interleukin-17
IFN-*γ*: Interferon-*γ*
RANKL: Receptor activator of nuclear factor-*κ*B ligand
TNF-*α*: Tumor necrosis factor-*α*
BMM: Bone marrow macrophage
M-CSF: Macrophage colony-stimulating factor
NC: Normal control
NS: Normal saline
HE: Hematoxylin and eosin
TRAP: Tartrate-resistant acid phosphatase
CEJ: Cemento-enamel junction
ABC: Alveolar bone crest
BV/TV: Bone volume per tissue volume
Ctsk: Cathepsin K
NFATc1: Nuclear factor of activated T cells 1
MMP-9: Matrix metallopeptidase 9
GAPDH: Glyceraldehyde-3-phosphate dehydrogenase
SASP: Senescence-associated secretory phenotype.

## Supplementary Materials

Supplementary Materials Figure S1: the number of TRAP-positive osteoclasts (A) and the area of TRAP-positive osteoclasts (B) were counted and presented (mean ± standard deviation, *n* = 3). ^#^*p* < 0.05 compared with the control group; ^&^*p* < 0.05 compared with the IL-17+IFN-*γ* group. Figure S2: in condition 2, BMMs were cultured with M-CSF (30 ng/ml) for 3 days, and on day 3, 20 ng/ml RANKL together with IL-17 (1 ng/ml) or/and IFN-*γ* (0.2 ng/ml) was added for 5 days. The number of TRAP-positive osteoclasts (A) and the area of TRAP-positive osteoclasts (B) were counted and presented (mean ± standard deviation, *n* = 3). ^#^*p* < 0.05 compared with the control group; ^&^*p* < 0.05 compared with the IL-17+IFN-*γ* group. Figure S3: effects of IL-17 (0.1, 10 ng/ml) or/and IFN-*γ* (0.02, 2 ng/ml) on the expression of osteoclastogenic genes *in vitro*. The mRNA expression of osteoclast-related genes (A) c-Fos, (B) NFATc1, (C) Ctsk, (D) MMP-9, and (E) TRAP was detected with different concentrations of IL-17 or/and IFN-*γ* using RT-qPCR. Data were standardized to GAPDH expression and shown as a fold change relative to the control group (mean ± standard deviation, *n* = 3). ^#^*p* < 0.05 compared with the control group; ^&^*p* < 0.05 compared with the IL-17+IFN-*γ* group. Table S1: primers used for RT-qPCR.

## References

[1] Pihlstrom B. L., Michalowicz B. S., Johnson N. W. (2005). Periodontal diseases. *Lancet*.

[2] Preshaw P. M. (2008). Host response modulation in periodontics. *Periodontology 2000*.

[3] Michaud D. S., Fu Z., Shi J., Chung M. (2017). Periodontal disease, tooth loss, and cancer risk. *Epidemiologic reviews*.

[4] Eke P. I., Dye B. A., Wei L. (2015). Update on prevalence of periodontitis in adults in the United States: NHANES 2009 to 2012. *Journal of Periodontology*.

[5] Franceschi C., Bonafe M., Valensin S. (2000). Inflamm-aging. An evolutionary perspective on immunosenescence. *Annals of the New York Academy of Sciences*.

[6] Childs B. G., Durik M., Baker D. J., van Deursen J. M. (2015). Cellular senescence in aging and age-related disease: from mechanisms to therapy. *Nature Medicine*.

[7] Franceschi C., Campisi J. (2014). Chronic inflammation (inflammaging) and its potential contribution to age-associated diseases. *The Journals of Gerontology. Series A, Biological Sciences and Medical Sciences*.

[8] Acharya P., Talahalli R. R. (2019). Aging and hyperglycemia intensify dyslipidemia-induced oxidative stress and inflammation in rats: assessment of restorative potentials of ALA and EPA + DHA. *Inflammation*.

[9] Ebersole J. L., Graves C. L., Gonzalez O. A. (2016). Aging, inflammation, immunity and periodontal disease. *Periodontology 2000*.

[10] Biwas I., Rezaie A. R. (2018). Vascular inflammation in aging. *Aging (Albany NY)*.

[11] Armitage G. C. (2000). Development of a classification system for periodontal diseases and conditions. *Northwest Dentistry*.

[12] Jacinto T. A., Meireles G. S., Dias A. T. (2018). Increased ROS production and DNA damage in monocytes are biomarkers of aging and atherosclerosis. *Biological Research*.

[13] Xia S., Zhang X., Zheng S. (2016). An update on inflamm-aging: mechanisms, prevention, and treatment. *Journal of Immunology Research*.

[14] Bartek J., Hodny Z., Lukas J. (2008). Cytokine loops driving senescence. *Nature Cell Biology*.

[15] Fessler J., Raicht A., Husic R. (2017). Novel senescent regulatory T-cell subset with impaired suppressive function in rheumatoid arthritis. *Frontiers in Immunology*.

[16] Thomas R., Wang W., Su D. M. (2020). Contributions of age-related thymic involution to immunosenescence and inflammaging. *Immunity & Ageing*.

[17] Alves A. S., Bueno V. (2019). Immunosenescence: participation of T lymphocytes and myeloid-derived suppressor cells in aging-related immune response changes. *Einstein (Sao Paulo)*.

[18] Hienz S. A., Paliwal S., Ivanovski S. (2015). Mechanisms of bone resorption in periodontitis. *Journal of Immunology Research*.

[19] Ohlrich E. J., Cullinan M. P., Seymour G. J. (2009). The immunopathogenesis of periodontal disease. *Australian Dental Journal*.

[20] Cheng W. C., Hughes F. J., Taams L. S. (2014). The presence, function and regulation of IL-17 and Th17 cells in periodontitis. *Journal of Clinical Periodontology*.

[21] Gaffen S. L., Hajishengallis G. (2008). A new inflammatory cytokine on the block: re-thinking periodontal disease and the Th1/Th2 paradigm in the context of Th17 cells and IL-17. *Journal of Dental Research*.

[22] Chen X. T., Tan J. Y., Lei L. H., Chen L. L. (2015). Cytokine levels in plasma and gingival crevicular fluid in chronic periodontitis. *American Journal of Dentistry*.

[23] Dutzan N., Abusleme L. (2019). T helper 17 cells as pathogenic drivers of periodontitis. *Advances in Experimental Medicine and Biology*.

[24] Eskan M. A., Jotwani R., Abe T. (2012). The leukocyte integrin antagonist Del-1 inhibits IL-17-mediated inflammatory bone loss. *Nature Immunology*.

[25] Dutzan N., Kajikawa T., Abusleme L. (2018). A dysbiotic microbiome triggers TH17 cells to mediate oral mucosal immunopathology in mice and humans. *Science translational medicine*.

[26] Jovanovic D. V., Di Battista J. A., Martel-Pelletier J. (1998). IL-17 stimulates the production and expression of proinflammatory cytokines, IL-beta and TNF-alpha, by human macrophages. *The Journal of Immunology*.

[27] Lees J. R. (2015). Interferon gamma in autoimmunity: a complicated player on a complex stage. *Cytokine*.

[28] Song L., Tan J., Wang Z. (2019). Interleukin 17A facilitates osteoclast differentiation and bone resorption via activation of autophagy in mouse bone marrow macrophages. *Molecular Medicine Reports*.

[29] Gao Y., Grassi F., Ryan M. R. (2007). IFN-gamma stimulates osteoclast formation and bone loss in vivo via antigen-driven T cell activation. *The Journal of Clinical Investigation*.

[30] Cheng J., Liu J., Shi Z. (2012). Molecular mechanisms of the biphasic effects of Interferon-*γ* on osteoclastogenesis. *Journal of Interferon & Cytokine Research*.

[31] Huang W., O'Keefe R. J., Schwarz E. M. (2003). Exposure to receptor-activator of NFkappaB ligand renders pre-osteoclasts resistant to IFN-gamma by inducing terminal differentiation. *Arthritis Research & Therapy*.

[32] Yang X., Zhang H., Wang J., Zhang Z., Li C. (2015). Puerarin decreases bone loss and collagen destruction in rats with ligature-induced periodontitis. *Journal of Periodontal Research*.

[33] Pan W., Wang Q., Chen Q. (2019). The cytokine network involved in the host immune response to periodontitis. *International Journal of Oral Science*.

[34] Kitami S., Tanaka H., Kawato T. (2010). IL-17A suppresses the expression of bone resorption-related proteinases and osteoclast differentiation via IL-17RA or IL-17RC receptors in RAW264.7 cells. *Biochimie*.

[35] Adamopoulos I. E., Chao C. C., Geissler R. (2010). Interleukin-17A upregulates receptor activator of NF-*κ*B on osteoclast precursors. *Arthritis Research & Therapy*.

[36] Ke D., Fu X., Xue Y. (2018). IL-17A regulates the autophagic activity of osteoclast precursors through RANKL-JNK1 signaling during osteoclastogenesis in vitro. *Biochemical and Biophysical Research Communications*.

[37] Kim J. W., Lee M. S., Lee C. H. (2012). Effect of interferon-*γ* on the fusion of mononuclear osteoclasts into bone-resorbing osteoclasts. *BMB Reports*.

[38] Salvioli S., Monti D., Lanzarini C. (2013). Immune system, cell senescence, aging and longevity--inflamm-aging reappraised. *Current Pharmaceutical Design*.

[39] Oates T. W., Graves D. T., Cochran D. L. (2002). Clinical, radiographic and biochemical assessment of IL-1/TNF-alpha antagonist inhibition of bone loss in experimental periodontitis. *Journal of Clinical Periodontology*.

[40] Johnson R. B., Serio F. G., Dai X. (1999). Vascular endothelial growth factors and progression of periodontal diseases. *Journal of Periodontology*.

[41] Ustun K., Erciyas K., Kisacik B. (2013). Host modulation in rheumatoid arthritis patients with TNF blockers significantly decreases biochemical parameters in periodontitis. *Inflammation*.

[42] Gorska R., Gregorek H., Kowalski J., Laskus-Perendyk A., Syczewska M., Madalinski K. (2003). Relationship between clinical parameters and cytokine profiles in inflamed gingival tissue and serum samples from patients with chronic periodontitis. *Journal of Clinical Periodontology*.

[43] Pesic G., Jeremic J., Nikolic T. (2017). Interleukin-6 as possible early marker of stress response after femoral fracture. *Molecular & Cellular Biochemistry*.

[44] Li J. Y., D'Amelio P., Robinson J. (2015). IL-17A is increased in humans with primary hyperparathyroidism and mediates PTH-induced bone loss in mice. *Cell Metabolism*.

[45] DeSelm C. J., Takahata Y., Warren J. (2012). IL-17 mediates estrogen-deficient osteoporosis in an Act1-dependent manner. *Journal of Cellular Biochemistry*.

[46] Duque G., Huang D. C., Dion N. (2011). Interferon-*γ* plays a role in bone formation in vivo and rescues osteoporosis in ovariectomized mice. *Journal of Bone and Mineral Research*.

[47] Tang M., Tian L., Luo G., Yu X. (2018). Interferon-gamma-mediated osteoimmunology. *Frontiers in Immunology*.

[48] Tu E., Ang D. K., Bellingham S. A. (2012). Both IFN-*γ* and IL-17 are required for the development of severe autoimmune gastritis. *European Journal of Immunology*.

[49] Doodes P. D., Cao Y., Hamel K. M. (2010). IFN-gamma regulates the requirement for IL-17 in proteoglycan-induced arthritis. *Journal of Immunology*.

[50] Wang Z., Zhong J., Tan J., Shen Y., Chen L. (2021). TRAF6/ERK/p38 pathway is involved in interleukin-17-mediated autophagy to promote osteoclast precursor cell differentiation. *Zhejiang Da Xue Xue Bao. Yi Xue Ban*.

[51] Li J., Zeng M., Yan K., Yang Y., Li H., Xu X. (2020). IL-17 promotes hepatocellular carcinoma through inhibiting apoptosis induced by IFN-*γ*. *Biochemical and Biophysical Research Communications*.

[52] Li J., Yan K., Yang Y., Li H., Wang Z., Xu X. (2019). Interleukin-17 promotes mouse hepatoma cell proliferation by antagonizing interferon-*γ*. *Nan Fang Yi Ke Da Xue Xue Bao*.

