# Supplementary material for: Inflamm-Aging-Related Cytokines of IL-17 and IFN-γ Accelerate Osteoclastogenesis and Periodontal Destruction
Source: J Immunol Res. 2021 Aug 4;2021:9919024. doi: 10.1155/2021/9919024 (PMC8357511; doi:10.1155/2021/9919024)
Supplement: Supplementary Materials — Figure S1: the number of TRAP-positive osteoclasts (A) and the area of TRAP-positive osteoclasts (B) were counted and presented (mean ± standard deviation, n = 3). #p < 0.05 compared with the control group; &p < 0.05 compared with the IL-17+IFN-γ group. Figure S2: in condition 2, BMMs were cultured with M-CSF (30 ng/ml) for 3 days, and on day 3, 20 ng/ml RANKL together with IL-17 (1 ng/ml) or/and IFN-γ (0.2 ng/ml) was added for 5 days. The number of TRAP-positive osteoclasts (A) and the area of TRAP-positive osteoclasts (B) were counted and presented (mean ± standard deviation, n = 3). #p < 0.05 compared with the control group; &p < 0.05 compared with the IL-17+IFN-γ group. Figure S3: effects of IL-17 (0.1, 10 ng/ml) or/and IFN-γ (0.02, 2 ng/ml) on the expression of osteoclastogenic genes in vitro. The mRNA expression of osteoclast-related genes (A) c-Fos, (B) NFATc1, (C) Ctsk, (D) MMP-9, and (E) TRAP was detected with different concentrations of IL-17 or/and IFN-γ using RT-qPCR. Data were standardized to GAPDH expression and shown as a fold change relative to the control group (mean ± standard deviation, n = 3). #p < 0.05 compared with the control group; &p < 0.05 compared with the IL-17+IFN-γ group. Table S1: primers used for RT-qPCR. [file 9919024.f1.zip › Table S1.docx]

Table S1. Primers used for RT-qPCR

| Gene | Primer sequence | Length |
| --- | --- | --- |
| mouse TRAP | Forward 5'-CACTCCCACCCTGAGATTTGT-3' Reverse 5'-CCCCAGAGACATGATGAAGTCA-3' | 145bp |
| mouse Ctsk | Forward 5'-CTCGGCGTTTAATTTGGGAGA-3' Reverse 5'-TCGAGAGGGAGGTATTCTGAGT-3' | 164bp |
| mouse NFATc1 | Forward 5'-GGAGAGTCCGAGAATCGAGAT-3' Reverse 5'-TTGCAGCTAGGAAGTACGTCT-3' | 102bp |
| mouse c-Fos | Forward 5'-CGGGTTTCAACGCCGACTA-3' Reverse 5'-TTGGCACTAGAGACGGACAGA-3' | 166bp |
| mouse MMP-9 | Forward 5'-CTGGACAGCCAGACACTAAAG-3' Reverse 5'-CTCGCGGCAAGTCTTCAGAG-3' | 145bp |
| mouse GAPDH | Forward 5'-GACTTCAACAGCAACTCCCAC-3' Reverse 5'-TCCACCACCCTGTTGCTGTA-3' | 125bp |
| rat IL-6 | Forward 5'-CCAATTTCCAATGCTCTCCT-3' Reverse 5'-ACCACAGTGAGGAATGTCCA-3' | 182bp |
| rat IL-1β | Forward 5'-ATGAGAGCATCCAGCTTCAAATC-3' Reverse 5'-CACACTAGCAGGTCGTCATCATC-3' | 214bp |
| rat TNF-α | Forward 5'-TCGAGTGACAAGCCCGTAG-3' Reverse 5'-CAGCCTTGTCCCTTGAAGAG-3' | 185bp |
| rat GAPDH | Forward 5'-CAAGTTCAACGGCACAGTCAAGG-3' Reverse 5'-ACATACTCAGCACCAGCATCACC-3' | 123bp |

TRAP: tartrate resistant acid phosphatase; Ctsk: cathepsin K; NFATc1: nuclear factor of activated T cells 1; MMP-9: matrix metallopeptidase-9; GAPDH: glyceraldehyde-3-phosphate dehydrogenase; IL-6: interleukin 6; IL-1β: interleukin 1 beta; TNF-α: tumor necrosis factor alpha.
